# The impact of boarding school on student development in primary and secondary schools: a meta-analysis

**DOI:** 10.3389/fpsyg.2024.1359626

**Published:** 2024-03-28

**Authors:** Zhiyong Zhong, Yang Feng, Yongqi Xu

**Affiliations:** School of Education, Minzu University of China, Beijing, China

**Keywords:** boarding school, student development, meta-analysis, primary and secondary school students, effect size

## Abstract

As a long-established model of schooling, the boarding system is commonly practiced in countries around the world. Numerous scholars have conducted a great deal of research on the relationship between the boarding school and student development, but the results of the research are quite divergent. In order to clarify the real effects of boarding school on students’ development, this study used meta-analysis to quantify 49 (91 effect sizes) experimental or quasi-experimental studies on related topics at home and abroad. The results find that: (1) Overall, boarding school has no significant predictive effect on student development, with a combined effect size of 0.002 (*p* > 0.05); (2) Specifically, boarding school has a significant positive predictive effect on students’ cognitive development (*g* = 0.248, *p* < 0.001), a significant negative predictive effect on students’ affective and attitudinal development (*g* = −0.159, *p* < 0.05), and no significant predictive effect on students’ behavioral development (*g* = −0.115, *p* > 0.05) and physical development (*g* = −0.038, *p* > 0.05); (3) The relationship between the two is moderated by the school stage and the type of boarding school, but not by the instruments; (4) Compared with primary school students, senior high school students and urban boarding students, the negative predictive effect of boarding system on junior middle school students and rural boarding students is more significant. In addition, there are some limitations in the study, such as the limited number of moderator variables included, the results of the study are easily affected by the quality of the included literature, and the dimensionality of the core variable “student development” is not comprehensive enough. In the future, further validation should be conducted through in-depth longitudinal or experimental studies.

## Introduction

Boarding school, which began in British public schools, is a common form of schooling that provides students with accommodation and food, and integrates personal lives of students with their academic lives (Dong, 2012). In boarding schools, a relatively closed school management model is generally adopted, and dormitories, canteens and other related living facilities are equipped to meet the basic living needs of students. The boarding school, as a mode of schooling, not only has a relatively long history in the West, but also has been practiced in China for nearly 40 years or so, covering all stages from kindergarten to university. There has been a great deal of academic research around boarding school, mainly including studies on the functions of boarding school (White, 2004), the internal management problems of schools (Zhang, 2006), the impact of boarding school on the physical and mental development of students (Kahane, 1988), and the relationship between boarding school and families (Ben-David and Erez-Darvish, 1997). With the increasing size of boarding school and the younger age of boarding students, boarding school has become an important and unique part of the school system. In recent years, research on the boarding school has gradually shifted from exploring the value implications to promoting students’ development, such as the impact of boarding on students’ academic performance (Foliano et al., 2019) and the impact of boarding on students’ mental health (Yang and Yan, 2022). However, these studies only discuss the relationship between boarding school and one aspect of student development. Indeed, student development encompasses multiple aspects of the educational process and developmental content (Pan, 2019). At the same time, some studies have pointed out that although boarding school helps students accept multiculturalism, promote students’ socialization (White, 2004) and enhance students’ academic performance (Zhou and Xu, 2021), there are also some negative effects, such as affecting the formation of students’ personality (Schaverien, 2010) causing social and emotional distress to students (Kleinfeld and Bloom, 1977), and affecting physical development (Xu et al., 2014). So, how does boarding actually affect the overall development of students? Are there differences in the role of different aspects of student development in a boarding environment? It is not only a summary of the effectiveness of the boarding school that has been implemented for a long time, but also an important question that needs to be answered urgently in order to promote the normalization and under-aging of boarding school.

### The correlation between boarding school and the development of students

Many studies have centered on the impact of boarding school on student development at different school stages, types of boarding school and instruments. However, there are some differences in the findings of the studies, which are broadly divided into three categories.

The first view is that boarding school has a significant positive predictive effect on student development. On the one hand, boarding school increases and standardizes the study time of students by providing a collectivized learning and living environment (Yao et al., 2018), which in turn improves students’ academic achievement (Curto and Fryer, 2014; Behaghel et al., 2017; Foliano et al., 2019). At the same time, boarding school also reduces students’ undesirable behaviors, such as a decline in absenteeism (Martin et al., 2014), and has a positive impact on students’ cognitive development. A survey by the American Association of Boarding Schools (2013) found that 68% of boarding school students believed that boarding school had helped them improve self-discipline, maturity, independence, cooperative learning, and critical thinking. On the other hand, group home living increases contact between students and promotes emotional communication and companionship among peers (Martin et al., 2014; Bosmans and Kerns, 2015). This close peer relationship not only helps boarding students better adapt to school life (Segal, 2013) and enhance their ability to live independently (Ma, 2012), but also increases student satisfaction with school and life, and promotes the development of students’ healthy personality (Wu et al., 2011). In addition, good peer relationships also serve as role models that can continuously stimulate students’ motivation and promote their interest in learning (Kennedy, 2010). Multi-subject attachment theory suggests that the scope of the attachment relationship is not limited to the parent–child relationship, and that teachers, as one of the important attachment objects for boarding students, can to some extent “substitute for the parents” and “compensate” for the lack of parent–child relationship of boarding students (Verschueren and Koomen, 2012). Supported by the theory of humanities and sociology and with the help of students’ autobiographies, White (2004) also amply substantiated the important role of boarding school in the development of students.

The second view is that boarding school has a significant negative predictive effect on student development. First of all, boarding school adopts a relatively closed management mode, which weakens the influence of the family and society in the growth of students, and causes certain harm to the physical and mental development of students (Schaverien, 2010). Especially for younger students, they are more dependent on their families, so the role of family environment is more important for their socialization (Yan et al., 2013). Secondly, boarding school is strictly regulated and competition within schools is fierce (Yao et al., 2018). Coupled with the dilution of parent–child relationship, students lack effective emotional support (Ye and Pan, 2007). As a result, boarding students are more likely to develop aversion to studying, leading to a decline in academic performance (Lu and Du, 2010), which in turn leads to undesirable behaviors, such as truancy, school bullying and dropping out of school (Pfeiffer and Pinquart, 2014; Shi and Zhao, 2016). Finally, the boarding environment increases the density of interactions between students, which tends to produce the contagion of negative emotions among peers (Li and Lin, 2019). It usually manifests itself in the form of interpersonal hypersensitivity, accompanied by depression, anxiety, paranoia and various other negative emotions and psychological problems (Niknami et al., 2011; Mander et al., 2014).

The third view is that boarding does not show significant differences in learning goals, learning engagement and mental health of students (Li, 2007; Martin et al., 2014). On the one hand, although boarding students have more psychological problems at the time of admission, as they move up the grades, they become more resilient to school life and their psychological problems gradually decrease (Liu et al., 2004; Xiao et al., 2010). On the other hand, boarding students can only communicate with their parents by phone as well as at home on weekends, which can not only dilute parent–child conflicts, but also satisfy students’ psychology of freedom and independence. Therefore, it is conducive to the development of parent–child relationship (Shen, 2021). Additionally, the problem of parental attachment is mitigated due to the growing influence of teacher-student and peer relationships on students (Wu et al., 2021).

### Potential moderators of the association between boarding school and the development of students

Different school stages can affect the effectiveness of boarding school on student development. Most studies identify age characteristics as the main factor influencing students’ mental health (Papworth, 2014; Wang and Mao, 2015). Primary school boarding students are young and have an imperfect level of physical and mental development. When primary school students are faced with an unfamiliar living environment, they often experience psychological maladaptation and difficulties in interpersonal interactions (Wang, 2015). Due to their relatively complete physical and mental development, junior middle school boarding students have basically formed psychological qualities such as cooperation, self-discipline and freedom, and have a relatively favorable psychological environment. It further supports the negative effects of underage boarding on children’s emotions and socialization (Wang, 2015). In addition, research is more divergent when it comes to academic development. Some scholars believed that there is no significant difference in the impact of boarding school on the academic performance of students in different grades (Bozdoğan et al., 2014), and at the same time, boarding has the same degree of positive impact on students in all grades (Gao, 2017). However, some scholars used instrumental variable regression to show that boarding has a more significant impact on the academic performance of primary school students, but not on junior middle school students (Qiao and Di, 2014). Thus, the effect of boarding school on student development may be moderated by different school stages.

Different types of boarding school affect the effectiveness of boarding on student development. In general, boarding school can be categorized into rural boarding school and urban boarding school. Studies with rural boarding students concluded that boarding school has a positive impact on the academic performance of rural students (Gao, 2017), which is consistent with the findings of numerous studies (Du et al., 2010; Kennedy, 2010); but studies with urban boarding students found that urban boarding students have a significant advantage in academic performance (Xu, 2019) and a better psychological condition than rural boarding students (Luo, 2013). Compared to rural boarding students, urban boarding students have better access to social resources, boarding environment, faculty, and more advanced concept of family education (Tan, 2020). In summary, there are some differences in the impact that different types of boarding school have on student development.

In terms of instruments, standardized scales, standardized tests, and self-administered questionnaires are widely used at present. Therefore, they can be divided into two categories: standardized and non-standardized instruments. The use of different instruments may affect the effectiveness of boarding on student development. For example, a self-administered questionnaire, the Mental Health Questionnaire for Junior Middle School Students, was used to measure the mental health level of students, and the results showed that the mental health of boarding students is significantly higher than that of non-boarding students (Zhang, 2020); the results measured using the Diagnostic Test of Mental Health (MHT) is the opposite of the former, showing that the mental health of non-boarding students is significantly better than that of boarding students (Chen, 2016). It follows that the effect of boarding school on student development may be moderated by the instruments.

### Current study

In summary, the overall effect of boarding school on student development needs to be further tested. In addition, factors such as different school stages, types of boarding school, and instruments may moderate the relationship between boarding school and student development. Established research mainly discusses one aspect of student development and the findings are not consistent. Therefore, this study adopts the meta-analytical approach to integrate, evaluate and analyze the existing empirical studies on boarding school and student development in order to draw general and generalized conclusions.

## Materials and methods

### Data retrieval strategies

This study utilized a variety of sources to collect literature related to the impact of boarding school on student development over the past three decades, both domestically and internationally. Specifically, firstly, the foreign language databases “Web of Science,” “Springer” and “Google Scholar” were searched with “boarding school,” “boarding” and “effect” and “impact” as the subject words, and a total of 1,325 foreign language documents were obtained. Secondly, in the Chinese databases of “CNKI,” “Wanfang Data” and “VIP “, a total of 1,524 Chinese literature was obtained by searching “boarding” and “boarding school” as the titles. The date of the search was 21 October 2023.

### Inclusion criteria

**Figure 1:**
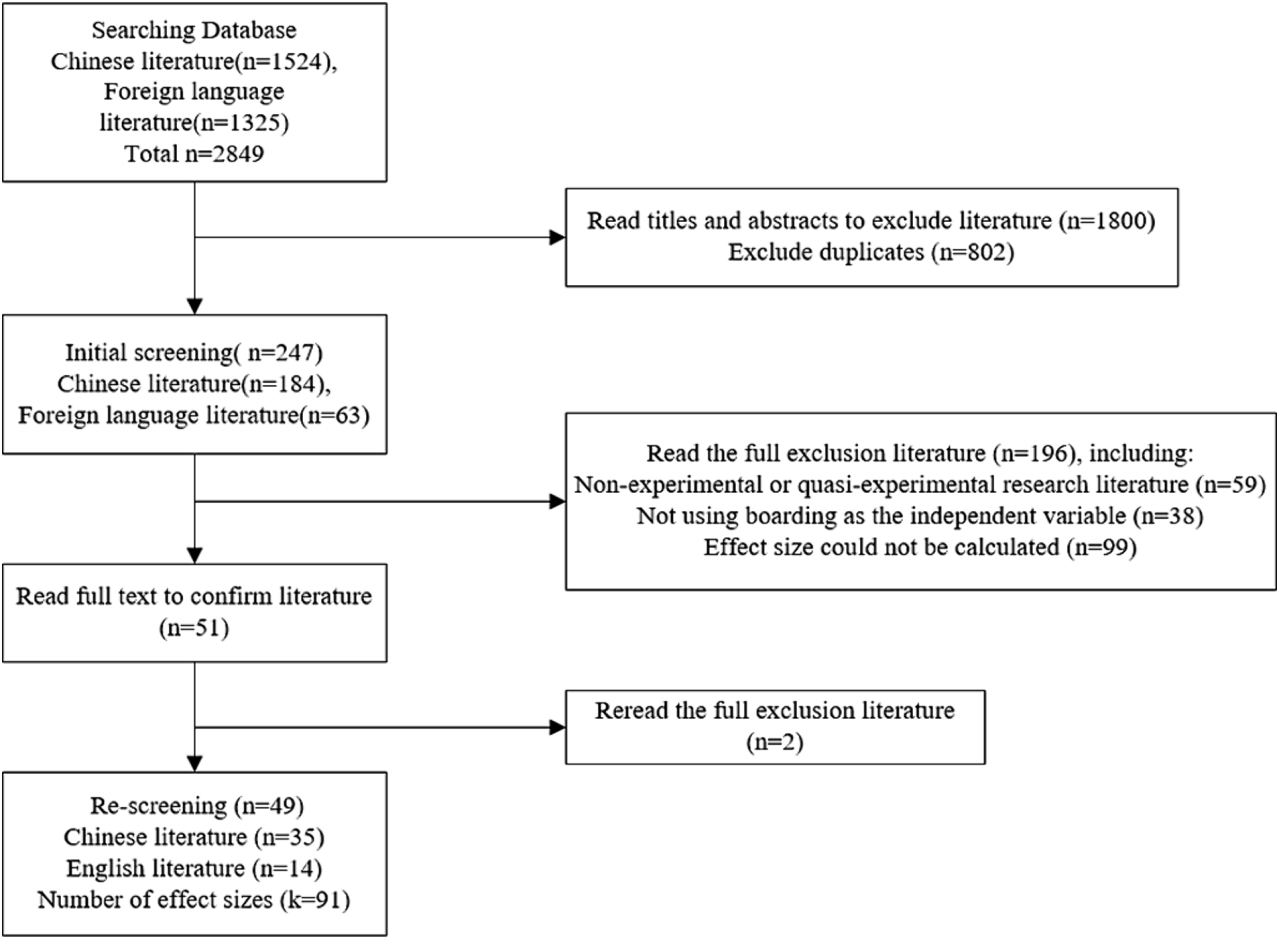
Flowchart of the inclusion protocol.

In this study, the Endnote20 literature management tool was used to screen the included literature according to the following criteria: (a) The topic of the study was the effect of boarding on students’ development; (b) The research subjects were primary and secondary school students; (c) The study needs to take boarding school as the independent variable; (d) The type of the study is an experiment or quasi-experiment comparing the differences in the development of boarding and non-boarding students, in which a single group of experiments need to provide pre- and post-tests data; (e) The study provides complete data that can calculate the effect size, such as the sample size (N), the mean (Means), the standard deviation (SD), or the *p*-value, t-value, and the correlation coefficient (r), and so forth; (f) Identical studies that had been published in a different format are excluded. After several rounds of literature screening and elimination of literature that did not meet the criteria, 49 papers were finally included and a total of 91 effect sizes were generated that could be used for meta-analysis. Among them, there were 35 articles in Chinese and 14 articles in foreign languages. The literature span from 1986 to 2023, but it was primarily focused on the last decade (Figure 1).

### Coding procedure

To further explore and analyze the impact of boarding school on students’ development, the key information was extracted and features coded from the included literature. In this study, 49 articles were independently coded by two coders to ensure reliability and consistency of the coding. There are three main aspects of coding:

The first is the basic information aspect of the literature, including the names of the authors, the time of publication, and data about the effect sizes.

Secondly, in terms of the dependent variable, this study used student development as the dependent variable. According to Benjamin Bloom’s Taxonomy of Educational Objectives (Anderson, 2009), student development is categorized into three dimensions: cognition, behavior, and affect and attitude. In addition, the dimension of physical development has been added in line with the boarding schools’ provision of food and accommodation. Among them, the cognitive dimension mainly consists of students’ academic performance and cognitive ability. Academic performance is a sufficient but not necessary condition for the cognitive development, thus a distinction is made here between academic performance and cognitive ability. The behavioral dimension includes both pro-social behaviors, etc., as well as problematic behaviors such as school bullying and absenteeism. The affective and attitudinal dimension includes students’ emotions, self-esteem, and motivation, etc. The physical development dimension includes the student’s BMI, nutrition, etc.

The third is the moderator variables, including three variables: school stage, the type of boarding school and instruments. First of all, the development of students is stage-specific and sequential, and the impact of choosing boarding at different school stages is also different, mainly including three stages: primary school, junior middle school and senior high school. Secondly, boarding schools can be divided into different types according to different classification criteria. In order to harmonize the definition of boarding school in domestic and foreign studies, this study mainly categorized boarding school into urban boarding school and rural boarding school according to geography. Finally, according to the degree of standardization of the instruments, they are divided into standardized and non-standardized instruments, where standardized instruments refer to the use of standardized questionnaires, scales, etc. to measure student development.

The included literature were coded according to the above characteristics, including author information, year of publication, dependent variable dimensions, school stages, school types, instruments, and effect size. The effect sizes d reported in the collected literature were transformed by the following equation: *g* = *d*[1−(3/(4 df−1)), df = n_1_ + n_2_-2. If the included studies did not report an effect size d, they were calculated from raw data such as sample size, mean, and standard deviation: d = (M1–M2)/Spooled, Spooled = [(n_1_–1) s_1_^2^ + (n_2_–1) s_2_^2^/n1 + n_2_-2]^1/2^. In addition, if the included studies did not fully report raw data such as sample size, mean, standard deviation, etc., they were transformed by the *χ*^2^ value, *F* value or t value of the raw data: *d* = 2[*χ*^2^/(N−*χ*^2^)^1/2^; *d* = 2/F (n_1_ + n_2_)/n_1_n_2_]^1/2;^ *d* = *t*/(n_1_ + n_2_/n_1_n_2_)^1/2^.

### Effect size

**Figure 2:**
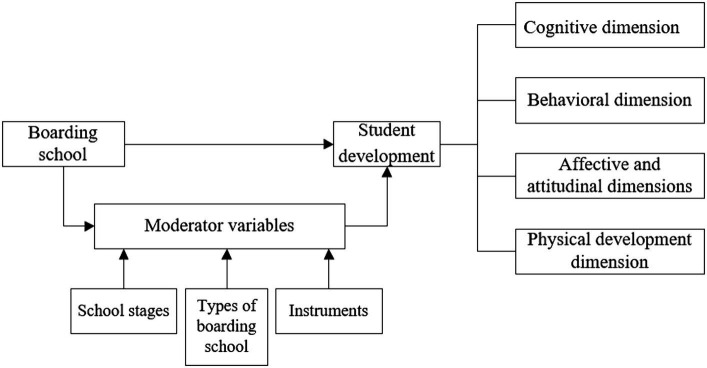
Meta-analytic framework diagram.

Due to the small sample size of this study, Hedges’ *g*-value was selected to measure the impact of boarding school on students’ development. According to Cohen’s criterion for judging the effect size: when the effect size is less than 0.2, its influence is small; when the effect size is more than 0.2 and less than 0.5, there is a moderate influence; when the effect size is more than 0.8, it has a large influence (Figure 2).

### Statistical analysis

The concept of meta-analysis was pioneered by Glass, an American psychologist. Meta-analysis, which aims to synthesize existing research, is a research process and systematic method for quantitatively combining and analyzing the effects of multiple conflicting studies on a given topic (Glass, 1976). In this study, the meta-analysis software Comprehensive Meta-Analysis Version 3.0 was used for data processing and analysis, and relevant data from the literature, such as the values of sample size, standard deviation, and mean, were entered into CMA for relevant calculations.

## Results

### Publication bias analysis

**Figure 3:**
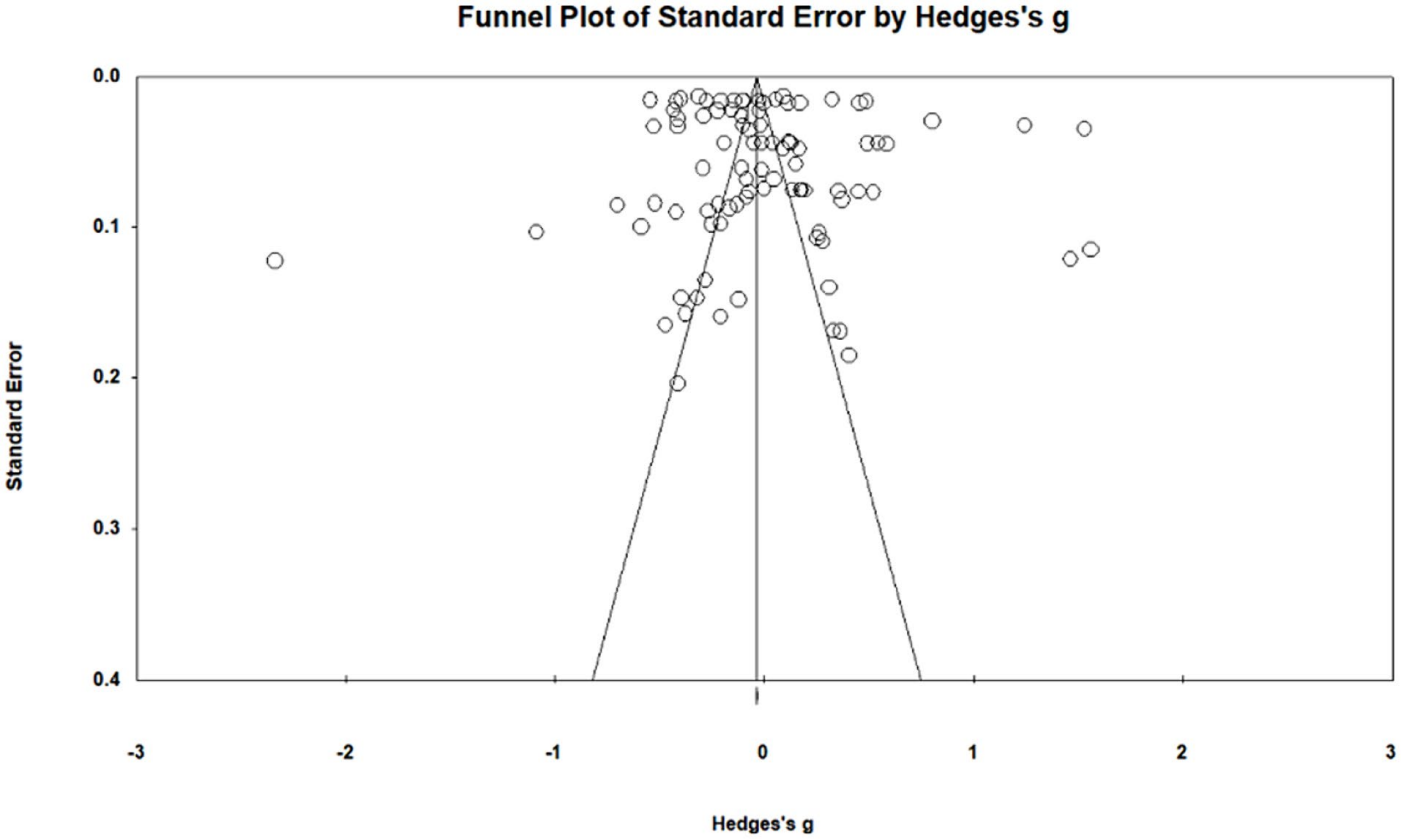
Funnel plot.

A publication bias analysis is first required before any specific data analysis of the sample literature can be conducted (Viechtbauer, 2007). Qualitative funnel plots and quantitative Egger’s were used for publication bias tests. Based on the funnel plot indicating (Figure 3) that the effect sizes of the study sample were focused on the upper middle region and more evenly distributed on both sides of the axis, it is initially judged that there is less likelihood of publication bias in the data. The study further utilized Egger’s method and the results of the data showed that *t* = 0.914 < 1.96 and *p* = 0.182 > 0.05, which satisfied the conditions of no publication bias (Egger et al., 1997). In summary, the results of meta-analysis were less likely to be biased for publication (Tables 1, 2).

**Table 1:** Coding table for meta-analytic variables.

Encoding	Coded fields	Code content
Dependent variable	Student development	CD: Cognitive dimension
		AA: Academic performance
		CA: Cognitive ability
		BD: Behavioral dimension
		EA: Affective and attitudinal dimensions
		PD: Physical development dimension
Moderator variables	School stage	P: Primary school
		M: Junior middle school
		H: Senior high school
	Type of boarding school	U: Urban boarding school
		R: Rural boarding school
	Instruments	Y: Standardized instruments
		N: Non-standardized instruments

**Table 2:** Summary of studies included in the meta-analysis.

References	Year	School stage	School type	Instruments	Dimension	Hedges’s g
Yao	2018	M	R	Y	AA/EA	0.096; −0.308
Yang	2023	M	R	N	BD	−0.408
Zhu	2019	P	R	Y	AA/EA	0.492; −0.49
Yang	2022	M	R	Y	EA/BD	−0.155; −0.429
Lu	2017	P	R	N	BD	−0.201
Ma 1	2013	P	U	Y	CA/EA	0.196; 0.358
Hou	2018	P	R	Y	AA/EA/BD	−0.139; −0.271; −0.095
Li 1	2017	P	R	Y	AA/EA	−0.218; −0.020
Li 2	2017	M	R	Y	AA/EA	0.117; 0.000
Xu	2019	M	U	Y	AA	−0.397
Zhang	2009	M	U	Y	EA	−0.519
Zhe Jiang1	1998	P	R	Y	CA	0.331
Zhe Jiang2	1998	P	R	Y	CA	0.365
Wu	2016	P	R	Y	EA/BD	−0.098; −0.020
Jiang	2020	P	R	Y	PD	−0.290; −0.102
Zhang	2014	M	U	Y	EA	−0.009
Wu 1	2017	P	U	N	AA	0.329; 0.059
Wu 2	2017	P	U	N	AA	0.459; 0.173
Zhang	2019	P	R	Y	EA	−0.416
Zan	2011	P	U	Y	EA	−0.126; −0.216
Yu	2021	P	U	Y	AA	0.093; 0.169
Yang	2016	M	U	Y	EA	−0.068
Xie	2016	P	R	Y	CA	−0.118
Wang	2016	H	R	Y	EA	−0.469
Sun	2019	H	R	N	BD	−0.392; −0.318
Shi	2016	H	U	Y	CA/EA	0.050; −0.082
Su	2019	M	R	Y	AA/EA	1.563; −1.091
Peng	2019	M	R	Y	EA	−0.699
Ma 2	2012	P	U	Y	AA	0.453; 0.524
Luo	2013	P	U	Y	AA	0.313
Liu	2017	P	U	Y	EA	−0.081
Liu	2018	M	R	Y	EA	−0.206
Lin	2020	P	R	Y	CA/BD/EA	−0.583; −0.249; −0.204
Feng	2023	M	U	N	EA	−2.337
Gao	2018	P	R	Y	EA	−0.540
Li	2007	H	U	N	AA/EA	0.376; −0.162
Yu 1	2023	M	R	Y	EA	−0.187
Yu 2	2023	M	R	Y	BD	0.128; 0.042; −0.049
Chen	2010	P	R	Y	EA	−0.263
Behaghel1	2015	M	R	Y	AA	0.176; 0.001
Behaghel2	2015	M	R	Y	EA	0.258; 0.136; 0.183
Foliano	2019	M	U	Y	AA	0.267; 0.282
Liu1	2020	M	R	Y	AA	0.588; 0.496
Liu2	2020	M	R	Y	EA/PD	−0.008; 0.126
Curto	2014	M	U	Y	AA	0.408; 0.155
Mutluer	2021	H	U	Y	EA	−0.371
Bin Tang	2020	P	R	Y	EA	−0.014; −0.098
Wang	2018	M	R	Y	AA/EA	−0.103; −0.286
Yang	2023	M	R	Y	EA	−0.523
Andersson	2013	P	U	Y	CA	1.465
Martin1	2014	M	U	Y	EA/BD	1.532; −0.408
Martin2	2014	M	U	Y	EA/BD	1.247; 0.807
Blau and Blau	2019	H	U	Y	EA	−0.278
Shirley Fisher	1986	M	U	N	EA	−0.409
Zhang	2016	M	R	N	PD/EA	0.121; 0.546
Chen	2018	P	R	N	PD	−0.067

### Heterogeneity analysis

There may be differences between the different studies included due to a number of factors. To avoid the inability to combine effect sizes due to the presence of heterogeneity in the study, the I^2^ statistic is generally used to determine the degree of heterogeneity in the sample, and thus to determine an effect model that is more appropriate for the study (Higgins, 2003). When *I*^2^ < 75%, a fixed effects model is used; when *I*^2^ > 75%, a random effects model is used. According to the test results, *I*^2^ = 99.309% > 75% and *Q* = 13020.408 (*p* < 0.001), the study had high heterogeneity (Table 3). Therefore, the random effect model would be chosen to analyze the effect of boarding school on student development in this study.

**Table 3:** Heterogeneity test results.

Effect model	*N*	*g*	95% CI		Homogeneity test			
			Lower	Upper	*I* ^2^	*Q*-value	*df*	*p*
Fixed effects model	91	−0.033	−0.039	−0.027				
Random effects model	91	0.002	−0.073	0.078	99.309	13020.408	90	0.000

### Main effects test

The results of the study indicated that boarding school was not a significant predictor of overall student development (*g* = 0.002, 95%CI = [−0.073, 0.078], *Z* = 0.053, *p* > 0.05). The study further examined the effect of boarding school on different dimensions of student development. According to the results of Table 4, the effect sizes from large to small were cognitive dimension (*g* = 0.248, *p* < 0.001) > affective and attitudinal dimension (*g* = |−0.159|, *p* < 0.05) > behavioral dimension (*g* = |−0.115|, *p* > 0.05) > physical development dimension (*g* = |−0.038|, *p* > 0.05). The results of the meta-analysis showed that boarding school had little effect on students’ overall development, but there were significant differences across the sub-dimensions. Specifically, boarding school has a moderate positive impact on students’ cognitive development and a small negative impact on students’ behavioral development, affective and attitudinal development, and physical development.

**Table 4:** The overall impact of boarding school on student development.

Student development	*N*	*g*	95% CI		Two-tailed test		Heterogeneity test within each group		
			Lower	Upper	*Z*	*p*	*Q*	df	*p*
Cognitive dimension	33	0.248	0.138	0.358	4.429	0.000			
Academic performance	26	0.249	0.135	0.362	4.293	0.000			
Cognitive ability	7	0.241	−0.193	0.675	1.090	0.276			
Behavioral dimension	13	−0.115	−0.284	0.054	−1.339	0.181	26.648	4	0.000
Affective and attitudinal dimension	40	−0.159	−0.292	−0.026	−2.339	0.019			
Physical development dimension	5	−0.038	−0.176	0.100	−0.544	0.587			

### Moderating effect test

Although the overall effect of boarding school on student development was small, there was significant heterogeneity in the effect size of different dimensions. Therefore, subgroup analyses are required. Moderating effect test was conducted using random effect model around different school stages, types of boarding school, and instruments.

### School stage

This study focuses on the impact of boarding school on the development of students in primary and secondary schools, so the school stages are coded into three groups: primary school, junior middle school and senior high school according to the current classification standards. Overall, there was a significant difference in the overall effect of different school stages on student development (*Q* = 6.895, *p* < 0.05), with the effect strengths between school stages in the following order: junior middle school (*g* = |−0.274|) > senior high school (*g* = |−0.158|) > primary school (*g* = 0.007) (Table 5). Specifically, there was a significant difference in the effect of boarding school on students’ physical development in the physical development dimension (*Q* = 13.481, *p* < 0.001). Among them, boarding school had a negative effect on the physical development of primary school students (*g* = −1.48, *p* < 0.05), while it had a positive effect on the physical development of junior middle school students (*g* = 0.123, *p* < 0.001). In addition, there was no significant difference in the cognitive dimension (*Q* = 0.144, *p* = 0.931), behavioral dimension (*Q* = 4.389, *p* = 0.111) and affective and attitudinal dimension (*Q* = 0.792, *p* = 0.673) (Table 6).

**Table 5:** The moderating effect of school stages on boarding school and student development.

School stage	*N*	*g*	95% CI		Two-tailed test		Heterogeneity test within each group		
			Lower	Upper	*Z*	*p*	*Q*	*df*	*p*
Primary school	22	0.007	−0.127	0.140	0.100	0.920			
Junior middle school	21	−0.274	−0.441	−0.107	−3.213	0.001	6.895	2	0.032
Senior high school	9	−0.158	−0.333	0.017	−1.771	0.077			

**Table 6:** The moderating effect of sub-dimension across school stages.

Dimension	School stage	*N*	*g*	95%	CI	Two-tailed test		Heterogeneity test within each group		
				Lower	Upper	*Z*	*p*	*Q*	*df*	*p*
	Primary school	14	0.231	0.092	0.369	3.259	0.001			
CD	Junior middle school	13	0.268	0.088	0.447	2.921	0.003	0.144	2	0.931
	Senior high school	2	0.209	−0.110	0.529	1.285	0.199			
	Primary school	4	−0.124	−0.220	−0.029	−2.554	0.011			
BD	Junior middle school	7	−0.046	−0.415	0.324	−0.242	0.809	4.389	2	0.111
	Senior high school	2	−0.355	−0.559	-0.150	−3.403	0.001			
	Primary school	14	−0.173	−0.289	-0.058	−2.933	0.003			
EA	Junior middle school	16	−0.296	−0.561	-0.031	−2.190	0.028	0.792	2	0.673
	Senior high school	5	−0.220	−0.352	-0.089	−3.279	0.001			
	Primary school	3	−0.148	−0.279	−0.016	−2.199	0.028	13.481	1	0.000
PD	Junior middle school	2	0.123	0.063	0.184	4.017	0.000			

### Type of boarding school

In this study, boarding schools were categorized into two types, rural boarding schools and urban boarding schools according to geography. Overall, there was a significant difference in the overall effect of different school types on student development (*Q* = 4.819, *p* < 0.05), with effect strengths in the following order: urban boarding (*g* = 0.126) > rural boarding (*g* = |−0.077|) (Table 7). Specifically, on the cognitive development dimension, there was a significant difference in the effect of boarding school on students’ cognitive development (*Q* = 5.903, *p* < 0.05). In this case, boarding school had no significant effect on the cognitive development of rural boarding students (*g* = 0.040, *p* < 0.05), while it produced a significant positive effect on the cognitive development of urban boarding students (*g* = 0.289, *p* < 0.001). In addition, there was no significant difference in the development of students across school types by boarding school on either the behavioral dimension (*Q* = 0.360, *p* = 0.549) or the affective and attitudinal dimension (Q = 0.251, *p* = 0.617) (Table 8).

**Table 7:** The moderating effect of school types on boarding school and student development.

School type	*N*	*g*	95% CI	Two-tailed test	Heterogeneity test within each group				
			Lower	Upper	*Z*	*p*	*Q*	*df*	*p*
Primary school	56	−0.077	−0.148	−0.006	−2.111	0.035			
Junior middle school	35	0.126	−0.041	0.293	1.484	0.138	4.819	1	0.028

**Table 8:** The moderating effect of sub-dimension across school type.

Dimension	School stage	*N*	*g*	95% CI		Two-tailed test		Heterogeneity test within each group		
				Lower	Upper	*Z*	*p*	*Q*	*df*	*p*
	Rural boarding school	11	0.040	−0.088	0.167	0.608	0.543			
CD	Urban boarding school	18	0.289	0.133	0.444	3.643	0.000	5.903	1	0.015
	Rural boarding school	11	−0.167	−0.272	−0.061	−3.097	0.002			
BD	Urban boarding school	2	0.199	−0.991	1.390	0.328	0.743	0.360	1	0.549
	Rural boarding school	20	−0.222	−0.334	−0.109	−3.870	0.000			
EA	Urban boarding school	15	−0.097	−0.571	0.376	−0.402	0.687	0.251	1	0.617

### Instruments

The reliability and scientificity of the findings of quantitative research will be affected to some extent by the research tool. As can be seen from the sample of literature, most of the studies used standardized tests or maturity scales to measure student development, while a small number of studies developed self-administered questionnaires to report students’ development. Therefore, the instruments were categorized into standardized and non-standardized instruments to further explore the moderating effect of instruments on the relationship between boarding school and student development. Overall, there was no significant difference in the overall impact of the different instruments on student development (*Q* = 0.128, *p* > 0.05). Specifically, on the behavioral dimension, there was a significant difference in the effect of boarding school on students’ behavioral development (*Q* = 4.274, *p* < 0.05). In particular, there was no significant negative effect of boarding school on students’ behavioral development when standardized instruments were used (*g* = −0.029, *p* > 0.05), while boarding school had a significant negative effect on students’ behavioral development when non-standardized instruments were used (*g* = −0.319, *p* < 0.001; Table 9). In addition, there were no significant differences between the boarding school on the cognitive dimension (*Q* = 0.004, *p* = 0.951), the affective and attitudinal dimension (*Q* = 0.198, *p* = 0.657), and the physical development dimension (*Q* = 0.498, *p* = 0.481) (Table 10).

**Table 9:** The moderating effect of instruments on boarding school and student development.

Instruments	*N*	*g*	95% CI		Two-tailed test		Heterogeneity test within each group		
			Lower	Upper	*Z*	*p*	*Q*	*df*	*p*
Standardized instruments	20	−0.081	−0.187	0.026	−1.489	0.137			
Non-standardized instruments	14	−0.117	−0.284	0.050	−1.373	0.170	0.128	1	0.720

**Table 10:** The moderating effect of sub-dimension across instruments.

Dimension	Instruments	*N*	*g*	95% CI		Two-tailed test		Heterogeneity test within each group		
				Lower	Upper	*Z*	*p*	*Q*	*df*	*p*
	Standardized instruments	7	0.265	−0.039	0.569	1.707	0.088			
CD	Non-standardized instruments	5	0.275	0.120	0.431	3.480	0.001	0.004	1	0.951
	Standardized instruments	9	−0.029	−0.255	0.196	−0.253	0.800			
BD	Non-standardized instruments	4	−0.319	−0.477	−0.162	−3.975	0.000	4.274	1	0.039
	Standardized instruments	10	−0.277	−0.427	−0.126	−3.600	0.000			
EA	Non-standardized instruments	3	−0.732	−2.736	1.271	2.743	0.474	0.198	1	0.657
	Standardized instruments	3	−0.086	−0.335	0.163	0.269	0.788			
PD	Non-standardized instruments	2	0.025	−0.159	0.210	−0.678	0.498	0.498	1	0.481

## Discussion

### The association between boarding school and the development of students

Compared with non-boarding school, boarding school has a smaller effect on student development (*g* = 0.002, *p* > 0.05), which supports the third view that there is no significant predictive effect of boarding on student development (Xiao et al., 2010; Martin et al., 2014; Sparks, 2015). The reason for this has much to do with the multidimensional concept of “student development.” There are many theories about the student development, the more typical ones are Social Learning Theory, Person-Environment Theory, Ecosystem Theory and so on. Together, these theories emphasize that student development is influenced by various factors, such as genetic, environmental, educational, and individuals. The boarding school provides students with a relatively closed learning environment, while integrating their studies and lives organically. In boarding schools, the extent to which students can be influenced by the environment in their interactions with it depends not only on the environment itself, but also on the students’ own initiative and motivation, school education, family environment and other factors (Du et al., 2010).

The results of the data show that boarding school reached a statistically significant level on cognitive development and affective and attitudinal development of the students. Therefore, the study only focuses on these two sub-dimensions for discussion. Boarding school has a positive and significant predictive effect on students’ cognitive development (*g* = 0.248, *p* < 0.001), which is consistent with previous findings (Kennedy, 2010; Lu and Du, 2010; Gao, 2017). Boarding life promotes the development of students’ self-awareness and increases their independence and self-discipline (Ma, 2012). These positive psychological qualities can be transferred to students’ learning, which in turn promotes the development of their cognitive abilities (TABS, 2023). Boarding school presents a negative and significant predictive effect on students’ affective and attitudinal development (*g* = −0.159, *p* < 0.05), which provides evidence for the second view (Ye and Pan, 2007; Mander et al., 2014). When a student enters a boarding school, he or she will be faced with a completely new environment, as well as the stripping away of parental attachments. Attachment theory suggests that stable attachment relationships are critical for students’ academic, emotional, and social development (Granot and Mayseless, 2001), while parents are the most important attachment relationship in students’ development (Bosmans and Kerns, 2015). In addition, boarding schools often have a closed management model, which can easily lead to problems such as academic overload and depression among students (Schaverien, 2010).

### School stage as a moderator

The relationship between boarding school and student development is moderated by different school stages (*Q* = 6.895, *p* < 0.05). Among them, boarding school has a significant negative effect on the development of junior middle school students, which may be related to the stage of physical and mental development that students are in (Wang, 2015). According to Piaget’s Cognitive-developmental Theory, junior middle school students are in the transition from the stage of concrete operations to the stage of formal operations, a period in which students shift from perceptual thinking to logical thinking. With the increasing difficulty of knowledge acquisition, it is a great challenge for students’ cognitive development. In addition, students’ physical functions and forms continue to develop and improve during this period, but their psychology is in a semi-mature and emotionally unstable stage. Some students will face a crisis of self-identity and a conflict of role confusion (Chen and Liu, 2019). Therefore, teachers should not only help students to stimulate their interest in learning, but also strengthen the support of families for students, and parents should be involved in students’ lives and learning.

### Type of boarding school as a moderator

According to the results of the data, the type of boarding school plays a moderating role between boarding school and student development (*Q* = 4.819, *p* < 0.05). Among them, rural boarding school has a negative effect on student development, which supports the views of Chen et al. (2018), Lu et al. (2017), Jiang and Xu (2020), and others. The result that urban boarding school has a positive effect on student development supports Luo (2013), Xu (2019), Blau and Blau (2021) and others. The main reason for the disparity lies in the economic differences between urban and rural areas. From the students’ point of view, rural boarding students are more likely to come from rural areas, where their families are economically limited and their parents are generally less educated. From the perspective of schools, urban boarding schools have better accommodations, hardware facilities, and teachers than rural boarding schools (Chen and Qi, 2010). Thus, it can be seen that boarding schools create variability in student development through differences in student population and level of schooling. In order to change the negative impact of the boarding school on rural students, the most important thing is to increase the total amount of financial input, and the gap between urban and rural areas is essentially an economic development gap. In addition, it is necessary to constantly expand the sources of funding to ensure the effective operation of the rural boarding school.

### Instruments as a moderator

There is no significant difference in the effect of the instruments on student development under the boarding condition, which suggests that the relationship between boarding school and student development is not moderated by the instruments (*Q* = 0.128, *p* > 0.05), but it is still of some analytical value. First, in terms of the specific effect size of the instruments, the effect size of using standardized instruments is smaller than that of non-standardized instruments. Although this difference does not reach the statistically significant level, it reflects the development trend of the two, that is, the measurement results of the non-standardized instruments are inflated compared with the standardized instruments. This is because standardized instruments are usually designed to be rigorous and preset the results within a certain range; whereas non-standardized instruments are usually a form of self-assessment and are more subjective, with flexible and open-ended results. Therefore, it can be presumed that standardized instruments are more realistic and reliable. Secondly, in terms of the scientific validity of the instruments, although the non-standardized instruments have not been recognized by the academic community and tested in practice like the mature standardized instruments, the operational procedures have been strictly followed and their reliability and validity tests have been tested, thus guaranteeing the scientificity and effectiveness of the instruments. This may also be one of the reason why the between-group effect failed to reach a statistically significant level.

### Limitations and future directions

The study used a meta-analytic approach to systematically analyze the effects of boarding school on the overall development of primary and secondary school students as well as on different sub-dimensions. In addition, the study explored the moderating effects of different school stages, types of boarding school and instruments. However, there are some limitations to this study. First of all, the number of moderating variables included is limited. There are many factors that affect student development, such as gender, family economic situation, peer relationships, etc., and more moderating variables should be included in the future. Secondly, the results of the study are based on the literature sample, which will be affected by factors such as the quality of the literature sample, the sample size and the research period. Finally, student development is a comprehensive and multidimensional concept that should also include the development of students’ skills, literacy, information literacy, etc. (Pan, 2019). Therefore, in the future, the validity of the findings of this study should be further verified by adopting a more scientific and comprehensive dimensionalization of the core concept of “student development.”

## Conclusion

This study utilized a meta-analytic research methodology to explore the impact of boarding school on student development in primary and secondary schools. The results showed that boarding school had no significant predictive effect on students’ overall development, but it was a significant positive predictor of cognitive development and a significant negative predictor of affective and attitudinal development. The relationship between boarding school and student development was also moderated by the stage and type school. The conclusions of the study provide some reference significance for the subsequent theoretical research, and provide new insights and suggestions for the implementation and improvement of the boarding school in practice.

## Data availability statement

The original contributions presented in the study are included in the article/supplementary material, further inquiries can be directed to the corresponding author.

## Author contributions

ZZ: Conceptualization, Methodology, Writing – review & editing. YF: Writing – original draft. YX: Project administration, Supervision, Writing – review & editing.

## References

[ref1] Anderson L. W. (2009). Bloom’s Taxonomy of Educational Objectives (Jiang, X. P, Luo, J. J, & Zhang, Q. M.). Bei jing: Wai yu jiao xue yu yan jiu chu ban she.

[ref2] Behaghel L. Chaisemartin C. Gurgand M. (2017). Ready for boarding? The effects of a boarding school for disadvantaged students. Am. Econ. J-Appl Econ. 9, 140–164. doi: 10.1257/app.20150090

[ref3] Ben-David A. Erez-Darvish T. (1997). The effect of the family on the emotional life of Ethiopian immigrant adolescents in boarding schools in Israel. Resid. Treat. Child. Yo. 15, 39–50. doi: 10.1300/j007v15n02_04

[ref4] Blau R. Blau P. (2021). Identity status, separation, and parent-adolescent relationships among boarding and day school students. Resid. Treat. Child. Yo. 38, 178–197. doi: 10.1080/0886571X.2019.1692757

[ref5] Bosmans G. Kerns K. A. (2015). Attachment in middle childhood: Progress and prospects. New Dir. Child Adolesc. Dev. 2015, 1–14. doi: 10.1002/cad.20100, PMID: 26086124

[ref6] Bozdoğan A. E. Günaydın E. Okur A. (2014). An examination of secondary school students’ academic achievement in science course and achievement scores in performance assignments with regard to different variables: a boarding school example. Particip. Educ. Res. 1, 95–105. doi: 10.17275/per.14.13.1.2

[ref7] Chen S. C. (2016). The relationship between mental health, social support and subjective well-being of boarding school students. Doctoral dissertation Qinghai Normal University.

[ref8] Chen Q. Chen Y. Zhao Q. (2018). Impacts of boarding on primary school students’ mental health outcomes – instrumental-variable evidence from rural northwestern China. Econ. Hum. Biol. 39:100920. doi: 10.1016/j.ehb.2020.100920, PMID: 32919377

[ref9] Chen Qi. & Liu R. D. (2019). Dang Dai Jiao Yu Xin Li Xue. Beijing: Beijing shi fan da xue chu ban she

[ref10] Chen W. H. Qi Y. B. (2010). Survey and analysis of the “rural boarding system project” in Guizhou Province. J. Anhui Agric. Sci. *38*, 1503–1506. doi: 10.13989/j.cnki.0517-6611.2010.03.162

[ref11] Curto V. E. Fryer R. G. (2014). The potential of urban boarding schools for the poor: evidence from seed. J. Labor Econ. 32, 65–93. doi: 10.1086/671798

[ref12] Dong S. H. (2012). Study on problem of boarding School in Rural Area in China. Master dissertation, Wuhan, HB, China: Central China Normal University.

[ref13] Du P. Zhao R. Y. Zhao D. C. (2010). A study on the academic achievement and school adaptability of rural elementary school boarding students from five provinces and autonomous regions in Western China. J. Educ. Stud. 6, 84–91. doi: 10.14082/j.cnki.1673-1298.2010.06.015

[ref14] Egger M. Smith G. D. Schneider M. Minder C. (1997). Bias in meta-analysis detected by a simple, graphical test. BMJ 315, 629–634. doi: 10.1136/bmj.315.7109.629, PMID: 9310563 PMC2127453

[ref15] Foliano F. Green F. Sartarelli M. (2019). Away from home, better at school. The case of a British boarding school. Econ. Educ. Rev. 73:101911. doi: 10.1016/j.econedurev.2019.101911

[ref16] Gao L. Y. (2017). Study on the impacting of the boarding system on students’ development of rural junior high schools. (Kaifeng, HN, China: Doctoral dissertation Henan University).

[ref17] Glass G. V. (1976). Primary, secondary, and meta-analysis of research. Educ. Res. 5:3. doi: 10.2307/1174772

[ref18] Granot D. Mayseless O. (2001). Attachment security and adjustment to school in middle childhood. Int. J. Behav. Dev. 25, 530–541. doi: 10.1080/01650250042000366

[ref19] Higgins J. P. (2003). Measuring inconsistency in meta-analyses. BMJ 327, 557–560. doi: 10.1136/bmj.327.7414.557, PMID: 12958120 PMC192859

[ref20] Jiang N. Xu J. Q. (2020). The impact of boarding on the Children’s health in rural China. J. Educ. Econ. 36:9.

[ref21] Kahane R. (1988). Multicode organizations: a conceptual framework for the analysis of boarding schools. Sociol. Educ. 61:211. doi: 10.2307/2112440

[ref22] Kennedy J. (2010). A case study of the influence of a small residential high school environment on academic success. (MN, USA: Doctoral Dissertation Walden University).

[ref23] Kleinfeld D. Bloom J. (1977). Boarding schools: effects on the mental health of eskimo adolescents. Am. J. Psychiatry 134, 411–417. doi: 10.1176/ajp.134.4.411, PMID: 842729

[ref24] Li D. L. (2007). The study about the grade and psychological health of the boarders and day students. (Jinan, SD, China: Doctoral dissertation Shandong Normal University).

[ref25] Li C. H. Lin W. L. (2019). Are negative emotion contagious? --based on perspective of class social network. China. Econ. Q. 18, 597–616. doi: 10.13821/j.cnki.ceq.2019.01.09

[ref26] Liu C. J. Tian S. Y. Xun G. L. Jia Z. L. (2004). Comparative study on the mental health state between students in boarding and non-boarding senior middle school. Chin. J. Tissue Eng. Res. 8, 5782–5784. doi: 10.3321/j.issn:1673-8225.2004.27.019

[ref27] Lu K. Du Y. H. (2010). The effect of layout adjustment of rural schools on student achievement analysis based on the two-level value-added model. Tsinghua. J. Educ. 6:10. doi: 10.14138/j.1001-4519.2010.06.004

[ref28] Lu W. Song Y. Q. Liang J. (2017). An empirical study on student bullying in boarding schools in rural China. J. Beijing. China. Norm. Univ (Soc Sci). 62, 5–17. doi: 10.3969/j.issn.1002-0209.2017.05.001

[ref29] Luo X. R. (2013). Research on the mental health of urban boarding primary school students–survey and analysis based on a boarding primary School in Shanghai. (SH, China: Doctoral dissertation Shanghai Normal University).

[ref30] Ma X. Y. (2012). Comparative study on the learning adapt ability, mental health andacademic achievement between pupils in-boarding and non-boarding primary school. (HN, China: Doctoral dissertation Hunan Normal University).

[ref31] Mander D. Lester L. Cross D. (2014). The social and emotional wellbeing, and mental health implications for adolescents transitioning to secondary boarding school. J. Child Adolesc. Ment. Health 8, 131–140.

[ref32] Martin A. J. Papworth B. Ginns P. Liem G. A. (2014). Boarding school, academic motivation and engagement, and psychological well-being. Am. Educ. Res. J. 51, 1007–1049. doi: 10.3102/0002831214532164

[ref33] Niknami S. Zamani-Alavijeh F. Shafiee A. Seifi M. (2011). 208 comparison of psychological status of full boarding and day students in boarding schools. Asian J. Psychiatr. 4, S52–S53. doi: 10.1016/s1876-2018(11)60201-3

[ref34] Pan H. Y. (2019). Student development research in Chinese universities --a text analysis based on the self-evaluation report of the evaluation and evaluation of domestic universities. (Doctoral dissertation Dalian University of Technology.

[ref35] Papworth B. (2014). Attending boarding school: A longitudinal study of its role in students’ academic and non-academic outcomes. Doctoral dissertation The University of Sydney.

[ref36] Pfeiffer J. P. Pinquart M. (2014). Bullying in German boarding schools: a pilot study. School. Psychol. Int. 35, 580–591. doi: 10.1177/0143034314525513

[ref37] Qiao T. Y. Di L. (2014). The causal inference of Boarding’s effect in rural primary and secondary school education. J. Soc. Dev. 2:15.

[ref38] Schaverien J. (2010). Boarding school: the trauma of the ‘privileged’ child. J. Anal. Psychol. 49, 683–705. doi: 10.1111/j.0021-8774.2004.00495.x 15533198

[ref39] Segal C. (2013). Misbehavior, education, and labor market outcomes. J. Eur. Econ. Assoc. 11, 743–779. doi: 10.1111/jeea.12025

[ref40] Shen T. (2021). A study on the mechanism of the impact of boarding on children’s subjective well-being --a multi-case analysis based on rootedness theory. Child Study 34, 31–39.

[ref41] Shi Y. B. Zhao X. X. (2016). Which is more influential to rural dropout: parents’ migration for work or school boarding? --based on the empirical analysis of 1881 junior high school students from three Western provinces. J. Educ. Econ. 32, 78–83+90.

[ref42] Sparks S. D. (2015). Study: boarding schools don’t benefit all students. Educ. Week 34:5.

[ref43] TABS. The Association of Boarding Schools. (2023). Available at: https://www.tabs.org/.

[ref44] Tan L. (2020). *A* study on the relationship between self-worth, time management disposition and academic performance of urban and rural boarding and non-boarding pupils. (CQ, China: Doctoral dissertation Southwest University).

[ref45] Verschueren K. Koomen H. M. Y. (2012). Teacher–child relationships from an attachment perspective. Attach Hum. Dev. 14, 205–211. doi: 10.1080/14616734.2012.672260, PMID: 22537520

[ref46] Viechtbauer W. (2007). Publication bias in meta-analysis: prevention, assessment and adjustments. Psychometrika 72, 269–271. doi: 10.1007/s11336-006-1450-y

[ref47] Wang Y. N. (2015). The investigation on mental health of boarders in ethnic minority Ares of Sichuan Province. (Mianyang, SC, China: Doctoral dissertation Southwest University of Science and Technology).

[ref48] Wang S. T. Mao Y. Q. (2015). The impact of boarding on social-emotional competence of left-behind children: an empirical study in 11 provinces and autonomous region in Western China. J. Edu. Stud. 11:10. doi: 10.14082/j.cnki.1673-1298.2015.05.014

[ref49] White M. A. (2004). An Australian co-educational boarding school: a sociological study of Anglo-Australian and overseas students’ attitudes from their own memoirs. Int. Educ. J. 5, 65–78.

[ref50] Wu H. Y. Ma G. Y. Jin S. H. (2011). On the development of personality and sociality in rural elementary boarding schools. J. Hebei. Norm. Univ. 13:4.

[ref51] Wu M. Zhou X. R. Ye P. Q. Sun L. P. (2021). The influence of teacher-student relationship, peer relationship and parent-child relationship on the psychological capital among rural primary school boarders: a moderated mediation model. Chin. J. Clin. Psychol. 29, 230–235. doi: 10.16128/j.cnki.1005-3611.2021.02.003

[ref52] Xiao M. Ge Y. Cao C. G. (2010). Correlation study about emotional management ability and mental health of the boarding countryside civilian workers’ children. Chin. J. School Health 31:3. doi: 10.16835/j.cnki.1000-9817.2010.11.006

[ref53] Xu Z. Z. (2019). Study on the mathematical performance and lts influencing factors of boarders and non-boarders --based on six large-scale regional tests. Educ. Sci. Res. 30:6.

[ref54] Xu H. Q. Zhang Q. Li L. Zhang F. Pan H. Hu X. Q. . (2014). Eating habits and nutritional status among boarding students in nutrition improvement program for rural area. Chin. J. School Health 35:4.

[ref55] Yan C. P. Fan R. Du W. Chen H. H. Li Y. H. (2013). The security characteristics of rural young boarding pupils and its influencing factors. Chin. J. Behav. Med. Brain Sci. 22:3. doi: 10.3760/cma.j.issn.1674-6554.2013.09.022

[ref56] Yang P. Yan Z. Y. (2022). How boarding schools affect student mental health? J. East. China. Norm. Univ (Educ Sci). 40:16. doi: 10.16382/j.cnki.1000-5560.2022.08.007

[ref57] Yao S. Gao L. Y. (2018). Can large scale construction of boarding schools promote the development of students in rural area better? J. Educ. Econ. 34, 53–60.

[ref58] Ye J. Z. Pan L. (2007). A study of the emotional world of rural boarding primar school students. Educ. Sci. Res. 18:3.

[ref59] Zhang C. W. (2006). New exploration of rural boarding school operation model. Peoples. Educ. 57:2.

[ref60] Zhang D. S. (2020). Analysis on boarding and non-boarding students in junior MiddleSchool in Qianxi County. (HB, China: Doctoral dissertation Hebei Normal University).

[ref61] Zhou J. Y. Xu L. N. (2021). The effect of boarding on Students' academic achievement, cognitive ability and non-cognitive ability in junior high school. Educ. Sci. Res. 1, 53–59. doi: 10.3969/j.issn.1009-718X.2021.05.010

